# MicroRNA-184 inhibits cell proliferation and invasion, and specifically targets TNFAIP2 in Glioma

**DOI:** 10.1186/s13046-015-0142-9

**Published:** 2015-03-26

**Authors:** Zhe Cheng, Hang Zhou Wang, Xuetao Li, Zhiwu Wu, Yong Han, Yanyan Li, Guilin Chen, Xueshun Xie, Yulun Huang, Ziwei Du, Youxin Zhou

**Affiliations:** Neurosurgery & Brain and Nerve Research Laboratory, The First Affiliated Hospital of Soochow University, 188 Shizi Street, Suzhou, Jiangsu 215006 Peoples Republic of China

**Keywords:** miR-184, TNFAIP2, Proliferation, Invasion, Glioma

## Abstract

**Background:**

miRNA-184 is an oncogene in human hepatocellular carcinoma but acts as a tumor suppressor in tongue squamous cell carcinoma. Studies have shown that miR-184 was down-regulated in glioma and TNFα-induced protein 2 (TNFAIP2) was closely related to tumorigenesis. This study aimed to determine the functions of miR-184 in glioma and the mechanisms of miRNA-184-TNFAIP2 mediated glioma progression.

**Methods:**

Real-time reverse-transcription PCR detected expression of miR-184 and TNFAIP2. U87 and U251 cells were transfected with miR-184 mimic, inhibitor, or negative control miRNA, and their invasion abilities were assayed. Cellular proliferation was measured by the cell counting kit-8 assay. miR-184 effects on glioma cell apoptosis and cell cycle were assessed by flow cytometer. Biological information software have predicted that miR-184 could target TNFα-induced protein 2 (TNFAIP2), Which was further validated by Western blot and qRT-PCR in glioma cells. *In vivo*, U87 cells transduced with either lentiviral over-expressed miR-184 or control lentivirus were injected into nude mice subcutaneously and intracranial respectively.

**Results:**

Expression of miR-184 was significantly lower in glioma tissues and cell-lines compared to normal brain tissues. Protein and mRNA expression of TNFAIP2 were inversely correlated with miR-184 in glioma. *In vitro*, proliferation and invasion abilities were also decreased in U87 and U251 cells after transfection with miR-184 mimic. *In vivo*, the xenografted tumor size in the miR-184 overexpressing group were smaller than the miR-NC group. Concordantly, U87 and U251 cells transfected with miR-184 mimic had a higher apoptosis rate, triggering an accumulation of cells at the G0/G1 phase and decreased cells in S-phase.

**Conclusions:**

miR-184 could regulate TNFAIP2 expression and affected its translation in glioma. miR-184 could also inhibit glioma progression and might serve as a novel therapeutic target in glioma.

**Electronic supplementary material:**

The online version of this article (doi:10.1186/s13046-015-0142-9) contains supplementary material, which is available to authorized users.

## Introduction

Gliomas are the most common and lethal primary brain tumors in adults. Patients with newly diagnosed glioblastoma multiforme (GBM), the most malignant histological subtype of glioma, have a median survival period of approximately one year [1-3]. Despite comprehensive therapies including surgical resection, radiation, and chemotherapy, the final prognosis of glioma remains extremely bad [4]. The significant property of a glioma usually develops in the craniocerebral depths and always infiltrates into adjacent normal brain tissue, so complete resection is difficult finished and very dangerous for patients with gliomas [5]. Therefore, novel biological molecular therapies that inhibit tumor cell growth and invasion are urgently needed.

MicroRNAs are endogenous small noncoding RNAs with lengths of approximately 18–25 nucleotides. They regulate gene expression at the transcriptional and post-transcriptional level by completely or incompletely binding to the 3′-UTR (untranslated regions) of their target gene messenger RNA (mRNA) and by repressing the translation or promoting the degradation of the target gene to exert biological functions [6-8]. There is mounting evidence confirming that microRNAs play pivotal roles in tumorigenic processes including cellular differentiation, proliferation, angiogenesis, cell death, apoptosis, and invasion [9]. MicroRNAs will hopefully be applied in clinical cancer diagnosis, therapy, and prognosis [10]. miR-184 is particularly enriched in the brain and testes in humans and is located in region 25.1 on the q-arm of chromosome 15. Its corresponding transcript is comparatively small (84 bp) and is not encoded near other clustered miRNAs [11,12]. Recently, researchers have shown that miR-184 functions as a potential oncogene in human hepatocellular carcinoma by suppressing Sox7 expression [13]. The overexpression of miR-184 might play an onco-miRNA role in the anti-apoptotic and proliferation processes. The plasma expression levels of miR-184 were also associated with the presence of primary tumors and might be used as a novel cancer marker in tongue squamous cell carcinoma [14]. However, the high expression of miR-184 reportedly causes a decrease in cell numbers and increases apoptosis in neuroblastoma cell-lines [15]. In concordance with the previous results, Tivnan *et al*. have demonstrated that miR-184 mediated inhibition tumor growth and prolonged the survival time in an orthotopic murine model of neuroblastomas [16]. However, miR-184 functions both as oncogene and tumor suppressor in the development and progression of numerous cancers. For glioma, miR-184 exhibited a progression-associated down-regulation miRNA; Overexpression of miR-184 in A172 and T98G glioma cells significantly decreased cell viability and proliferation [17]. Besides, a study by Emdad et al. confirmed that miR-184 is down-regulated in human malignant glioma cells and tumor tissue as compared with their non-neoplastic counterpart [18]. The functions and exact mechanisms of miR-184 in glioma are poorly uderstood.

TNFα-induced protein 2 (TNFAIP2) maps to chromosome 14q32, encodes a 654 amino acid protein, belongs to the Sec6 family, is differentially expressed in capillary tube-like formation *in vitro*, and can be induced to express itself with a TNFα treatment [19]. TNFAIP2 was highly expressed in marrow from patients with acute myelogenous leukemia subtypes M0-M2 but was repressed in marrow cells from M3 patients. It was also a target gene of PML-RARs (promyelocytic leukemia-retinoic acid receptor α) in APL (acute promyelocytic leukemia) [20]. Recently, Chen *et al*. found that TNFAIP2 is highly expressed in tumor cells compared to adjacent normal tissue, is closely related to invasion and metastasis, and may serve as an independent prognostic indicator for nasopharyngeal carcinoma (NPC) [21]. A previous study showed that miR-184 directly targeted the 3′-UTR of TNFAIP2 using a dual-luciferase reporter assay in lung, head, and neck cancer cell-lines. The miR-184 binding site single nucleotide polymorphisms [SNP (rs8126 T > C)] in the 3′-UTR of TNFAIP2 modulated TNFAIP2 expression and contributed to susceptibility to squamous cell carcinoma of the head and neck (SCCHN) [22].

The expression and function of TNFAIP2 have not been studied in gliomas; the relationship between TNFAIP2 and miR-184 also has not been studied. This study confirmed that miR-184 was lower in glioma cell-lines and in 49 glioma specimens than in normal brain tissues. The up-regulation of miR-184 inhibited glioma cell proliferation and invasion and induced apoptosis. It also increased the cell population in the G0/G1 phase and reduced the percentage of U87 and U251 cells in the S phase. The expression of TNFAIP2 was higher in 81 glioma samples compared to noncancerous brain tissues. The results of this experiment implied that miR-184 might be a suppressor gene and conformed that miR-184 could target TNFAIP2 in gliomas.

## Materials and methods

### Human tissue samples

Tissue samples were obtained from March 2009 and September 2011 from the Department of Neurosurgery of the First Affiliated Hospital of Soochow University. There were a total of 81 glioma samples (29 cases low-grade WHO grades I and II, 52 cases high-grade WHO grades III and IV) and 8 non-neoplastic brain tissues obtained from patients who suffered severe cerebral injury. Their injuries required a reduction in increased intracranial pressure by removing a partial amount of normal brain tissue. The mean age of the patients at the time of surgery was 48.9 years for 31 women and 49.76 years for 50 men. There were 2 cases of pilocytic astrocytoma (grade I), 22cases of diffuse astrocytoma and 5 cases of pleomorphic xanthoastrocytoma (grade II), 19 cases of anaplastic astrocytoma and 5 cases of oligoastrocytoma (grade III), 28 cases of primary brain glioblastoma (grade IV). All tissue specimens were immediately collected and stored in liquid nitrogen after resection. This study was authorized by the local ethics committee of our hospital. All patients were informed of their participation in the study before surgery and gave their consent.

### Cells and cell culture

The glioma cell-lines U87, U373, U251, and A172 were purchased from the Cell Bank Type Culture Collection of the Chinese Academy of Sciences (Shanghai, China). U373 cells were retired from the ATCC catalogue, since similar characteristics were found between U373 and U251 cells. SHG44 and normal human astrocyte 1800 cells were conserved and provided by our Brain and Nerve Research Laboratory. All cell-lines were maintained in Dulbecco’s modified eagle’s medium (DMEM, Hyclone, Thermo Fisher Scientific, Waltham, MA, USA) supplemented with 100 U of penicillin/ml, 100 mg of streptomycin/ml, and 10% fetal bovine serum (FBS, Gibco) at 37°C under a humidified atmosphere of 5% CO2.

### Transfection

U87 and U251 cells were transfected with miR-184 mimic, miR-184 inhibitor, and their corresponding negative control (miR-NC and anti-NC) by Lipofectamine2000 (Invitrogen Inc., Carlsbad, CA, USA), which were designed and synthesized by Invitrogen. The sequences were as follows: miR-184 mimic, 5′-UGGACGGAGAACUGAUAAGGGUCCUUAUCAGUUCUCCGUCCAUU-3′; the negative control (miR-NC), 5′-UUCUCCGAACGUGUCACGUTTACGUGACACGUUCGGAGAATT-3′; miR-184 inhibitor, 5′-ACCCUUAUCAGUUCUCCGUCCA-3′; and the negative control (anti-NC), 5′-CAGUACUUUUGUGUAGUACAA-3′. Mock group was untreated by anything. To achieve stable transfectant overexpression of miR-184 and negative control oligonucleotide, pLenti-miR-184-GFP and pLenti-NC-GFP (Genechem Co., Ltd., Shanghai, China) were applied to transfect U87 cells.

### Quantitative RT-PCR

RNA was extracted using TRIzol reagent (Invitrogen Inc., Carlsbad, CA, USA), which was quatified by spectrophotometer . Only the RNA samples with 260/280 ratios of 1.8–2.0 were used for further investigation. The miR-184 expression level was measured using All-in-One™miRNA qRT-PCR Detection Kit (GeneCopoeia, Rockville, MD, USA) according to the instructions. Briefly, 100 ng RNA was used to synthesize cDNA in a 25 μl reaction system containing 5 μl 5× Reaction Buffer, 1 μl RTase Mix, and 1 μl 2.5 U/μl Poly A Polymerase. All reaction systems were incubated at 37°C for 60 min and 85°C for 5 min. The PCR conditions for miRNA quantification were as follows: 10 min at 95°C, then 40 cycles of 10 s at 95°C, 20 s at 62°C, and 30 s at 72°C. The relative level of TNFAIP2 mRNA was examined using SYBR green qRT-PCR (Applied LightCycler480). Subsequently, 2 μg of total RNA were reverse transcribed in a 20 μl reaction containing 10 units of M-MLV reverse transcriptase and 0.5 μg of oligo (dT) primer. A total of 2 μl of cDNA was used for qPCR. The expression level of GAPDH was used as an internal control for mRNAs , and the endogenous U6 snRNA as an internal control for miRNAs. The following primers were used: TNFAIP2 forward primer, 5′-CCTGCTCTCCCTACGC-3′, reverse primer, 5′-CGTCCAAGATGCTCCG-3′ [19]; and GAPDH forward primer, 5′-AACGGATTTGGTCGTATTG-3′, reverse primer, 5′-GGAAGATGGTGATGGGATT-3′. The PCR conditions for relative quantification were as follows: initial denaturation at 95°C for 5 min, then 40 cycles consisting of 95°C for 10 s, 60°C for 30 s, and 72°C for 30 s. The relative expression of each gene was calculated and normalized using the 2^−ΔΔCt^ method. Each sample was tested in triplicate.

### Cell proliferation, cell cycle, and cell apoptosis

Cell proliferation was measured by a cell counting kit-8 (CCK-8) (Beyotime, Shanghai, China). Cells (2 × 10^3^ per well) were seeded in a 96-well plate and incubated for 24 h. Then, the cells were transfected with miR-184 mimic, miR-184 inhibitor, or the negative control miRNA (Negative)at a final concentration of 50 nmol/L. CCK-8 (10 μl) was added to each well at 24, 48, and 72 h after transfection, and plates were incubated for 4 h at 37°C. Absorbance was measured at a wavelength of 450 nm. For cell cycle analysis, transfected cells were fixed in 75% ethanol at 4°C and stained with propidium iodide (PI). The cell cycle distribution was analyzed by flow cytometer (CYTOMICS, FC, 500 Beckman-coulter, CA, USA). Cell apoptosis was also detected using the Annexin V PE Apoptosis Detection Kit PE (eBioscience, San Diego, CA, USA) by flow cytometer. Cells transfected with miR-184 mimic, inhibitor, or negative control miRNA were collected and resuspended in 400 μl of 1× binding buffer containing 5 μl 7-AAD (7-amino-actinomycin D) and 5 μl PI at room temperature in the dark for 10–15 min. All experiments were performed in triplicate.

### Wounding healing and invasion assay

For the cell migration and cell invasion assay, all the cells (U87, U251) were used after transfection. For the cell motility assay, cells were seeded in six-well plates and cultured to 70-80% confluence. A 200 μl pipette tip was applied to generate a linear wound. The floating cells were rinsed with culture medium. Cells cultured for 48 h and were recorded under a microscope (Olympus, Tokyo, Japan) at 0, 6, 12, 24, and 48 h. For the cell invasion assay, Transwell chambers were covered with Matrigel (BD Bioscience, San Jose, CA, USA), and 1 × 10^5^ cells suspended in 100 μl serum-free medium were added to the upper chambers. The lower chambers were filled with 750 μl DMEM with 10% fetal bovine serum. After 48 h of incubation, non-invading cells on the upper surface of the membrane were removed using a cotton swab. The invaded cells were fixed in 4% formaldehyde for 30 min and stained with 0.1% crystal violet for 5 min. Then, cells were imaged and counted under a microscope. The number of cells that penetrated the Matrigel was counted from 6 randomly selected fields.

### Western blotting

Total protein was isolated by RIPA and quantified using a BCA (bicinchonininc acid) assay kit (Beyotime, Shanghai, China). Equal amounts of protein samples were separated by 12% SDS-PAGE and transferred into nitrocellulose membranes. After blocking, the membranes were incubated with primary antibodies overnight at 4°C. After washing, membranes were incubated with HRP-conjugated anti-rabbit secondary antibodies at a dilution of 1:4000 (Prosci Inc., Poway, CA, USA) at room temperature for 2 h. Then, the membranes were detected and recorded using ECL Western blotting detection reagents. The primary antibodies used were rabbit anti-TNFAIP2 at a dilution of 1:1000 (Santa Cruz Biotechnology, Santa Cruz, CA, USA) and rabbit anti-GAPDH at a dilution of 1:2000 (Abcam, Tokyo, Japan).

### Xenograft experiments

To select stably expressed cells, U87 cells were transfected with pLenti-miR-184-GFP or pLenti-NC-GFP. U87-miR-184 and U87-miR-NC cells were established and inoculated into nude mice (4 to 5 weeks old) in intracranial (1 × 10^5^) and subcutaneous (1 × 10^6^) fashions, respectively (n = 6 /group). Caliper measurements were performed to assess tumor growth. The size is represented by the width of subcutaneous tumor multiply the length of subcutaneous tumor (W*L). MRI (Magnetic Resonance Imaging) was also used to observe tumor growth. Each nude mouse was anesthetized by 200 μl 4% chloral hydrate, then scanned by Signa HDX model 3.0 T. MRI machine (GE., Detroit, USA). T2-weight images were acquired, and the following parameters were used: repetition time 2160.00 milliseconds, echo time 108.80 milliseconds, field of view 10 × 10 cm, and matrix 256 × 256. About 5 weeks post-implantation, the nude mice were euthanized and the tumors obtained. All mice experimental procedures were performed according to the First Affiliated Hospital of Soochow University policies.

### Immunohistochemistry

Formalin-fixed paraffin-embedded U87 intracranial tumors were cut into 6-μm-thick sections on a microtome. Immunohistochemical staining was performed according to standard procedures. Antigen retrieval was conducted in 0.1 M citrate buffer (pH 6.0) at 95°C for 16 min and cooled at 25°C for 1 h. After blocking, slides were incubated with primary antibodies TNFAIP2 (1:200, Santa Cruz Biotechnology, Santa Cruz, CA, USA), SOX2 (1:200, Boster Bioengineering Co., Wuhan, China) and ki-67 (1:200, Boster Bioengineering Co., Wuhan, China). Next, according to the manufacturer’s instructions, slides were treated with the Cell & Tissue Staining Kit HRP-DAB system (R&D Systems, Minneapolis, MN, USA). The immunohistochemical staining results were evaluated by two experienced pathologists.

### Statistical analysis

Statistical analyses were performed using GraphPad PRISM4.0 software (GraphPad, La Jolla, CA, USA) and SPSS version 13.0 (SPSS, Chicago, IL, USA). Experimental data are all presented as the means ± standard deviation (SD). Statistical analyses were performed using a two-tailed Student’s t-test or ANOVA and P < 0.05 was considered statistically significant.

## Results

### The expression level of miR-184 was low in 49 cases of glioma and 5 glioma cell-lines

**Figure 1:**
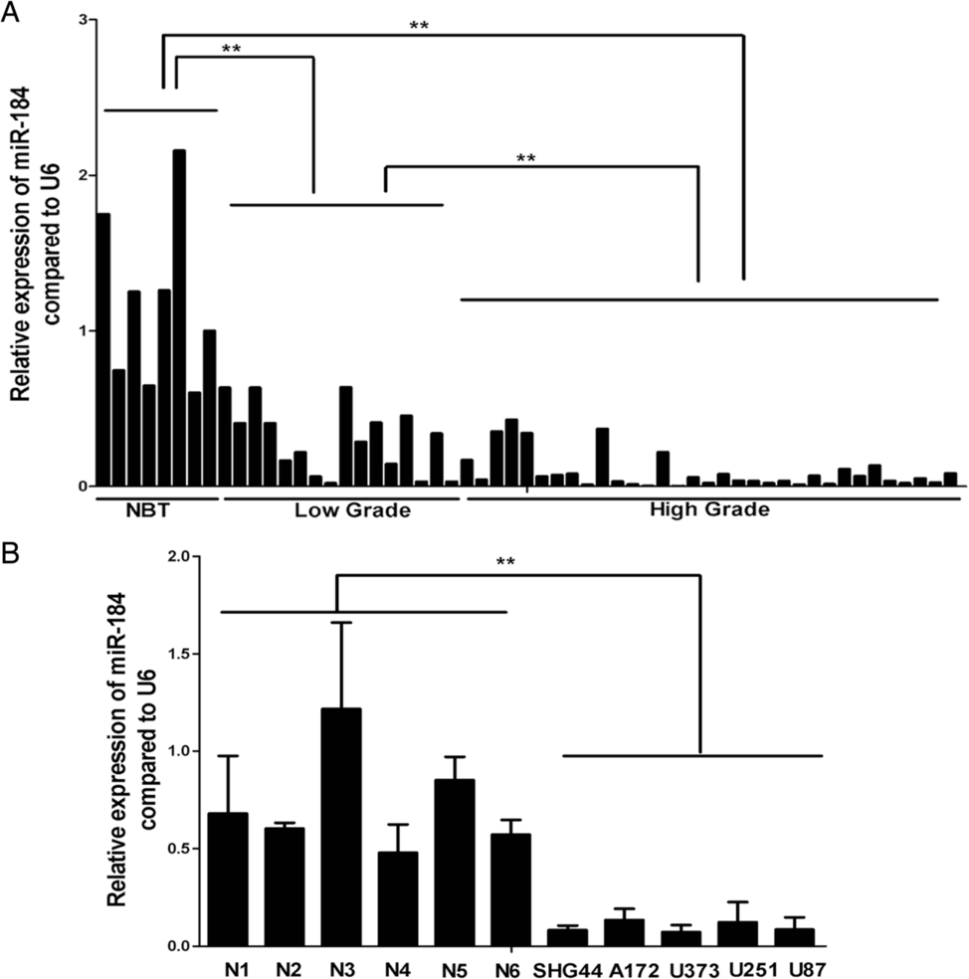
**miR-184 was down-regulated in human glioma tissues and glioma cell-lines. A**. shows the qRT-PCR analysis results of miR-184 expression in glioma tissues and normal brain tissues (NBT). It showed that miR-184 expression in 49 gliomas was markedly lower than in 8 non-cancerous brain tissues and decreased with the increasing degree of malignancy in gliomas (low grade vs high grade). U6 small nuclear RNA was used as an internal control. **B**. shows that miR-184 expression in 5 glioma cell-lines was down-regulated compared to 6 normal brain tissues (N1, N2, N3, N4, N5, N6) .U6 small nuclear RNA was used as an internal control. Data are shown as mean + s.d. (n = 3); *indicates P-value <0.05, **indicates P-value <0.01.

Previous studies have shown that miR-184 was down-regulated in glioma cell-lines and tissues and decreased with the increasing degree of malignancy [17,18], but too few cases were studied. To confirm the results of the present study, the number of glioma cases was increased to detect the expression of miR-184. Quantitative reverse transcriptase PCR (qRT-PCR) results demonstrated that miR-184 expression in 49 glioma tissues was markedly lower than in 8 noncancerous brain tissues and decreased with the increasing degree of malignancy in gliomas (low-grade vs high-grade, P < 0.01, Figure 1A, Table 1). Meanwhile, miR-184 expression was also examined in glioma cell-lines (U87, U251, U373, A72, SHG44). As demonstrated in Figure 1B, the expression of miR-184 was significantly reduced in 5 glioma cell-lines compared to 6 non-tumor brain tissues. These results suggested that low expression of miR-184 might be associated with the malignant glioma process and might act as a tumor suppressor in gliomas.

**Table 1:** **2** ^**-**ΔΔ**CT**^ **values of miR-184 expression in 57 cases (normalized to U6)**

**Noncancerous samples**	**Glioma samples**		**P value**
	**Low grade**	**High grade**	
**(n = 8)**	**(n = 16)**	**(n = 33)**	
1.1772 ± 0.55268			P_1_ = 0.003
	0.3057 ± 0.2197		P_2_ = 0.001
		0.0945 ± 0.31666	P_3_ = 0.002

2^-ΔΔCT^ values of miR-184 and U6 were detected by real-time quantitative PCR. P_1_(Noncancerous VS Low grade), P_2_(Noncancerous VS High grade), P_3_(Low grade VS High grade).

### The expression of TNFAIP2 was high in glioma cells and tissues

**Figure 2:**
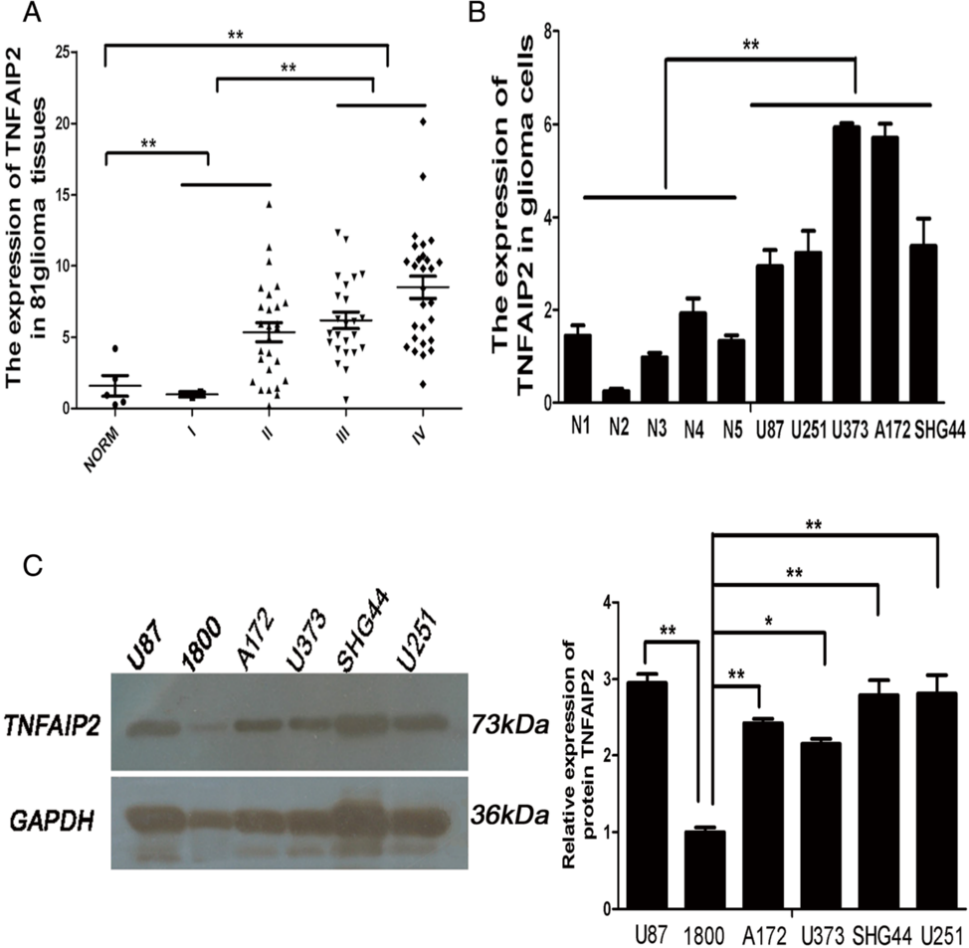
**TNFAIP2 expression was up-regulated in 81 glioma tissues and 5 glioma cells. A**. shows that the expression levels of TNFAIP2 in 81 human glioma tissues were higher than in 5 non-cancerous brain tissues normalized to GAPDH. TNFAIP2 expression was higher in high-grade gliomas (WHO grades III and IV) than in low-grade gliomas (WHO grades I and II). **B**. shows that the expression levels of TNFAIP2 mRNA in 5 glioma cell-lines were higher than in5 non-cancerous brain tissues. **C**. The left panel shows the Western blot results, which further confirmed that the expression level of TNFAIP2 protein was higher in glioma cell-lines than in normal human astrocyte 1800 cells. The right panel shows the relative expression of TNFAIP2 protein in glioma cells, as depicted in the histogram. Data are shown as mean + s.d. (n = 3); *indicates P-value <0.05, **indicates P-value <0.01.

TNFAIP2 expression and its correlation with miR-184 in gliomas have not been previously reported. QRT-PCR results showed that the mRNA expression level of TNFAIP2 was up-regulated in 5 glioma cell-lines compared to 5 normal brain samples (Figure 2B), and highly expressed in 81 glioma tissues (WHO grade I, 2 cases; II, 27 cases; III, 24 cases; and IV, 28 cases) compared to non-neoplastic brain tissues (Figure 2A, Table 2). Western blotting results further confirmed that the expression level of TNFAIP2 protein was higher in 5 glioma cell-lines than in normal human astrocyte 1800 cells (Figure 2C). Therefore, the expressions of miR-184 and TNFAIP2 were negatively correlated in gliomas.

**Table 2:** **2** ^**-**ΔΔ**CT**^ **values of TNFAIP2 mRNA expression in 86 cases (normalized to GAPDH)**

**Noncancerous samples**	**Glioma samples**		**P value**
	**Low grade**	**High grade**	
**(n = 5)**	**(n = 29)**	**(n = 52)**	
1.5905 ± 1.62443			P_1_ = 0.042
	5.0454 ± 3.55360		P_2_ = 0.001
		7.4272 ± 3.72806	P_3_ = 0.006

2^-ΔΔCT^ values of miR-184 and U6 were detected by real-time quantitative PCR. P_1_(Noncancerous VS Low grade), P_2_(Noncancerous VS High grade), P_3_(Low grade VS High grade).

### Up-regulation of miR-184 directly regulated the low expression of TNFAIP2 in human glioma cells

**Figure 3:**
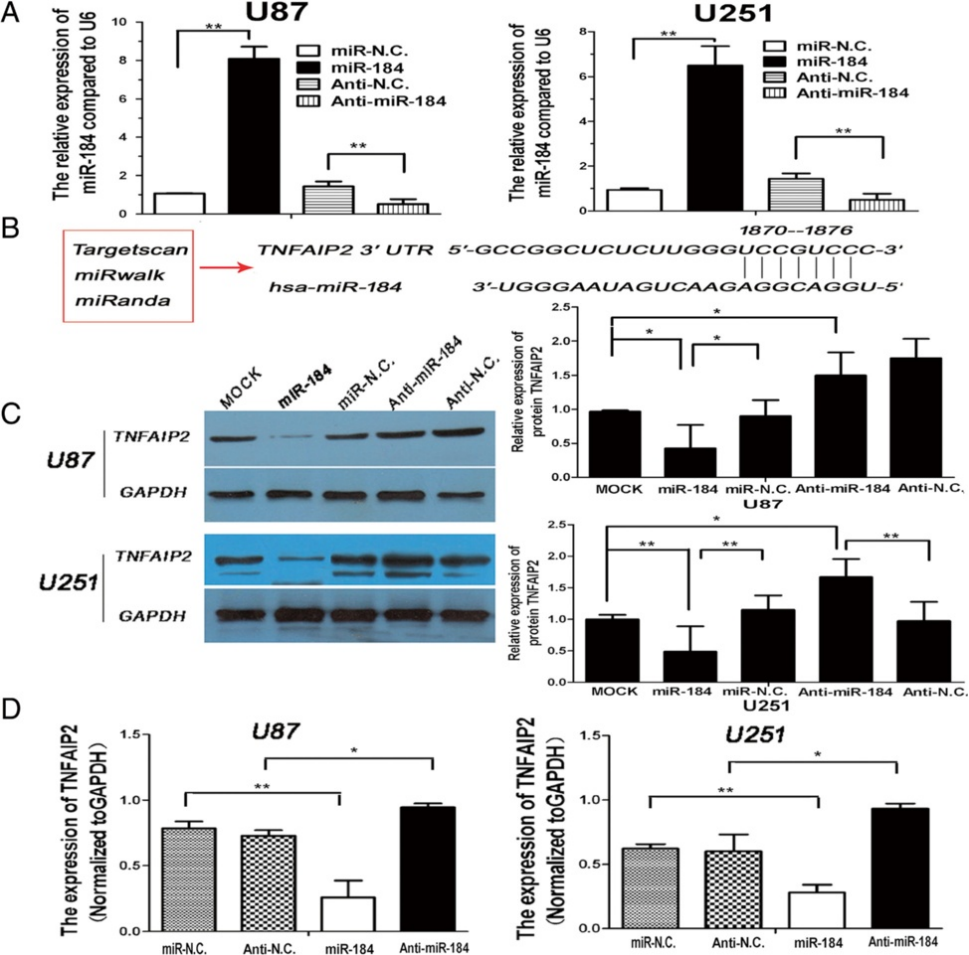
**The expression of miR-184 in U87 and U251 cells after transfection modulated the expression of TNFAIP2. A**. To keep a high transfection efficiency, qRT-PCR was used to detect the expression of miR-184 in U87 and U251 cells 48 h after transfection with miR-184 mimic, inhibitors, and the negative control miRNA. U6 was used as the loading control. **B**. shows the bioinformatic analysis using Targetscan, miRwalk, and miRanda, which predicted that miR-184 targeted TNFAIP2. **C**. shows the left panel, in which the Western blot analysis showed that miR-184 inhibited the expression of TNFAIP2 compared with the Mock group or the negative control miRNA group. The right panel shows the relative expression of TNFAIP2 protein in glioma cells, as depicted in the histogram. The protein expression levels were normalized to GAPDH. **D**. depicts the results of the qRT-PCR analysis, which showed that miR-184 inhibited the mRNA expression of TNFAIP2 in U87 and U251 cells compared with their corresponding negative control. GAPDH was used as the loading control. Data are shown as mean + s.d. (n = 3); *indicates P-value <0.05, **indicates P-value <0.01.

**Figure 4:**
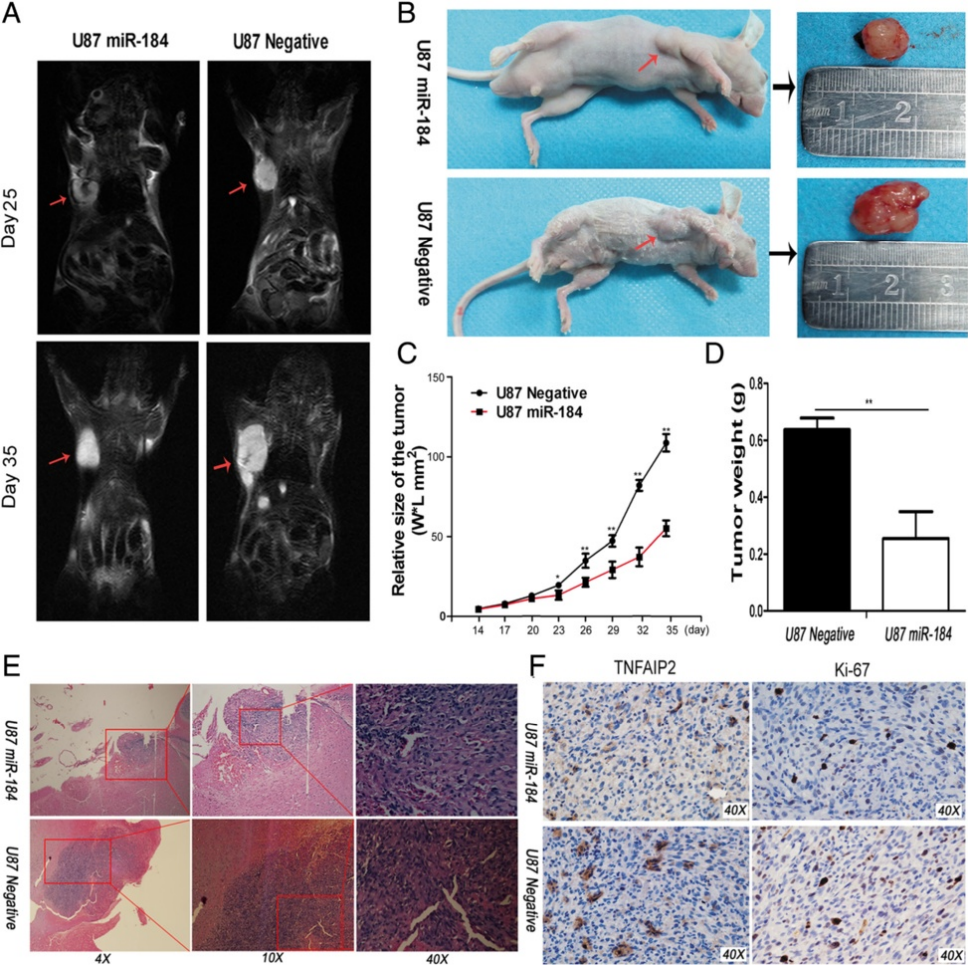
**miR-184 reduced glioma growth and TNFAIP2 expression** ***in vivo*** **in the mice model. A**. shows the T2-weighted MRI imaging of subcutaneous tumor growth at days 25 and 35 in U87-miR-184 and U87-Negative nude mice (red arrows indicate tumors). **B**.-**D**. show that miR-184 reduced glioma growth in the subcutaneous glioma nude mice model. **E**. shows the representative H&E staining, which revealed that miR-184 reduced glioma growth in the encephalic glioma nude mice model. **F**. shows that miR-184 reduced TNFAIP2 and ki-67 expression in an *in vivo* mice model. Data are shown as mean + s.d. (n = 3); *indicates P-value <0.05, **indicates P-value <0.01.

Biological information software (Targetscan, miRwalk, miRanda) have predicted that TNFAIP2 was one of the miR-184 target genes (Figure 3B) and have characterized TNFAIP2 as a direct target of miR-184 by a dual-luciferase reporter assay in lung cancer cells [22]. To examine whether miR-184 regulated TNFAIP2 expression in gliomas, U87 and U251 cells were infected with miR-184 mimic, inhibitor , negative control miRNA. Figure 3A shows that the expressions of miR-184 in U87 and U251 cells after transfection were detected by qRT-PCR. The miR-184 expression level was significantly increased after transfection with miR-184 mimic, while its expression was decreased after transfection with inhibitors, as compared to their corresponding negative control (miR-NC and anti-NC). TNFAIP2 was measured both by qRT-PCR and Western blotting. The data showed that the up-regulation of miR-184 led to the obviously down-regulated expression of TNFAIP2 and reduced the expression of miR-184 resulted in the significant up-regulation of TNFAIP2 (Figure 3C-D). Therefore, both TNFAIP2 mRNA and protein expressions were down-regulated by ectopic miR-184 in glioma cells. The immunohistochemical staining results also showed that the glioma xenografts of the U87-miR-184 group expressed less TNFAIP2 than the tumors in the U87-negative group (Figure 4F). These collective results suggested that TNFAIP2 is a genuine target of miR-184 in gliomas.

### Up-regulation of miR-184 reduced the invasion and migration of U87 and U251 cells

**Figure 5:**
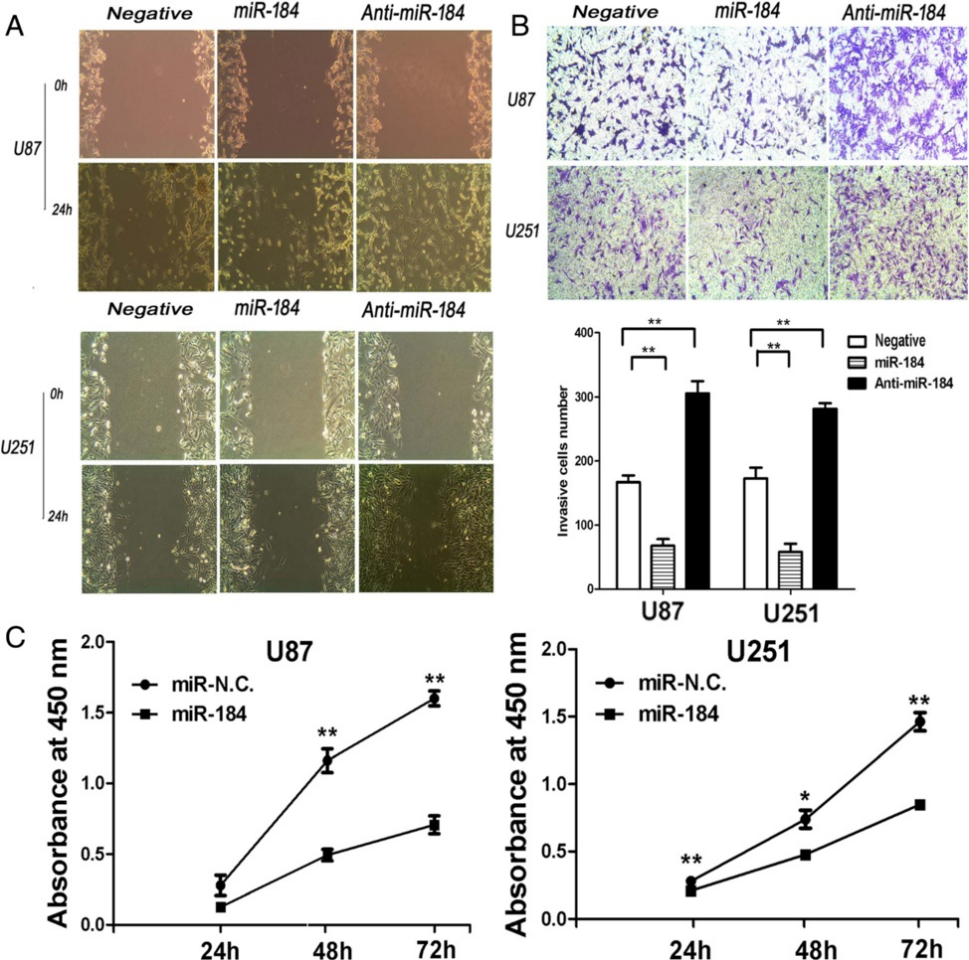
**Up-miR-184 reduced the invasiveness and growth of glioma cells. A**. Wound healing assay shows that the migration of U87 and U251 cells transfected with miR-184 mimics or inhibitor. The overexpression of miR-184 inhibited the wound closure speed of U87 and U251 cells compared with the Negative group or inhibitor group. **B**. Transwell assay shows the invasion of U87 and U251 cells transfected with miR-184 mimics or inhibitor. which revealed that the numbers of invasive cells were significantly reduced compared with the Negative group or inhibitor group. Each bar represents the mean values from three independent experiments. The invaded cells were counted from 6 random fields. **C**. shows the cell survival, which was determined by the CCK-8 assay. Cell proliferation was dramatically decreased in U251 and U87 cells after transfection with miR-184. Data are shown as mean + s.d. (n = 3); *indicates P-value <0.05, **indicates P-value <0.01.

To further study the invasion and migration ability of miR-184, Wound healing and Matrigel invasion assays were performed. The wound healing assay showed that miR-184 expression reduced the wound closure speed of U87 and U251 cells compared with negative control oligonucleotide (Negative) and anti-miR-184 groups. Contrarily, wound closure speed was improved by transfection with miR-184 inhibitor compared with Negative and miR-184 groups (Figure 5A). In accordance with the results of the wound healing assay, the Transwell matrix penetration assay showed that the overexpression of miR-184 markedly suppressed the invasiveness of U87 and U251 cells compared to Negative and anti-miR-184 groups (Figure 5B). These findings suggested that miR-184 inhibited the migration and invasion of U87 and U251 cells *in vitro*.

### Up-regulation of miR-184 suppressed glioma cell proliferation *in vitro* and *in vivo*, induced apoptosis, and inhibited the cell cycle

**Figure 6:**
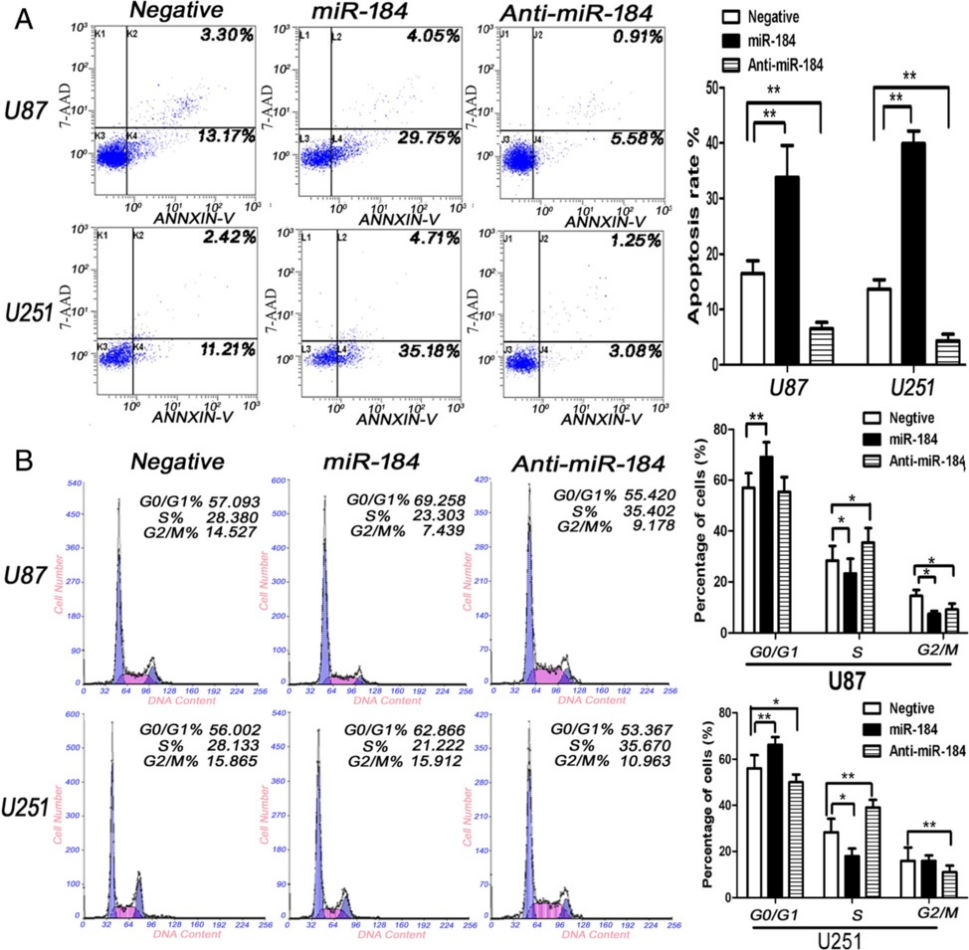
**miR-184 overexpression induced apoptosis and increased the cell population in the G0/G1 phase. A**. shows that miR-184 overexpression induced apoptosis in U87 and U251 cells and down-regulated of miR-184 reduced apoptosis compared with Negative group. The left panel shows representative pictures of up-miR-184, which increased the apoptosis of U87 and U251 cells. The right panel shows the relative number of apoptosis cells. **B**. shows the left panel, in which representative pictures show that miR-184 increased the cell population in the G0/G1 phase and reduced the percentage of cells in the S phase compared with the Negative group. The right panel shows the quantitative data of cells in different cell cycle phases. Data are shown as mean + s.d. (n = 3); *indicates P-value <0.05, **indicates P-value <0.01 VS Mock.

As shown in Figure 5C, the CCK-8 assay was performed to detect the effects of miR-184 on U87 and U25 cells growth *in vitro*. The data demonstrated that the growth curve was significantly inhibited in the miR-184 mimic-transfected cells compared to the negative control groups, and there was a significant difference between the control (miR-NC)group and miR-184 group (Table 3). To illustrate the mechanisms how miR-184 modulates glioma cell growth, flow cytometry was applied to detect the effects of miR-184 on cell apoptosis and the cell cycle. The results indicated that the proportion of apoptosis cells transfected with miR-184 was significantly higher than in the anti-miR-184 group and the negative group (Figure 6A). Moreover, miR-184 induced U87 and U251 cells cycle arrest and could increase the cell population in G0/G1 phase and reduce the percentage of cells in S-phase compared to the negative group (Figure 6B). The role of miR-184 in growth was next explored *in vivo*. The stable expression of U87 cells of miR-184 or miR-NC were established through a lentivirus infection method and inoculated into nude mice in intracranial and subcutaneous, respectively. The immunohistochemical (IHC) results of SOX2 was used to ensure that U87 cells grow and form an intracranial tumor (Additional file 1). As shown in our experiments, tumors were efficiently suppressed in miR-184 group compared to negative group (Figure 4A). Representative intracranial H&E stainings of xenograft tumors in nude mice showed that miR-184 reduced glioma growth, as shown in Figure 4E. The tumor size of the xenografts further confirmed that the U87 miR-184 overexpression group showed slower tumor growth than the U87 negative control group *in vivo*. The IHC results of the nude mice intracerebral transplantation tumors also showed that U87 cells transfected with miR-184 had reduced ki-67 expression compared to the control group. The average expression rate of Ki-67 is only 33% in U87 -miR-184 group, 75% in U87 negative group (p < 0.01) (Figure 4F).

**Table 3:** **Absorbance were measured at 450 nm of U87 and U251**

**Time**	**U87**		**P value**	**U251**		**P value**
	**miR-NC**	**miR-184**		**miR-NC**	**miR-184**	
24 h	0.2798 ± 0.1250	0.1266 ± 0.0175	0.167	0.2790 ± 0.0086	0.2093 ± 0.0072	0.000
48 h	1.1602 ± 0.1443	0.4917 ± 0.0705	0.002	0.7397 ± 0.1150	0.4777 ± 0.0438	0.021
72 h	1.5997 ± 0.0920	0.7066 ± 0.1102	0.000	1.4623 ± 0.1166	0.8495 ± 0.0570	0.001

U87and U251 cells were transfected with miR-184 mimic or miR-NC, and cell counts were detected using CCK-8. The difference between two groups was analyzed by the Studentns t-test.

## Discussion

MiRNAs directly modulate and inversely regulate different genes via inducing mRNA cleavage or translation repression and function as oncogenes or tumor suppressors depending on the target genes [23,24]. Accumulated evidence shows that miRNAs play a pivotal role in the development of the malignant phenotype of glioma cells, including cell survival, proliferation, angiogenesis, differentiation, and stem cell generation [25]. The effects of miR-184 on malignant progression are debated because it can act as a tumor promoter or suppressor in some solid tumors [14,13,26]. Previous experimental studies have demonstrated that miR-184 acted as a modulator in the malignant progression of gliomas [17] and was expressed lower in glioma cells and tissues [18]. The collective data provided sufficient indication that miR-184 might serve as a tumor suppressor gene in gliomas. Gliomas are characterized by high invasion, migration, and proliferation abilities [27], so it is necessary to study the molecular mechanisms of miR-184 modulate gliomas. Even though previous studies have confirmed that miR-184 was down-regulated in gliomas and decreased with the increasing degree of malignancy, the number of cases used was too limited. The present study increased the glioma cases to further confirm the expression and function of miR-184 in gliomas.

The present study’s results were in accordance with those from previous studies. The miR-184 expression levels were determined in 49 glioma tissues and 5 glioma cell-lines by qRT-PCR analysis, which displayed a remarkable down-regulation of miR-184 in gliomas compared to 6 non-tumor brain tissues. Also, miR-184 down-regulation might be linked to glioma development. Wound healing and Matrigel invasion assays were performed to conclude that the overexpression of miR-184 markedly suppressed the invasiveness of U87 and U251 cells in comparison with the negative control group. miR-184 performed an important function in glioma invasion. *In vitro*, the proliferation of glioma cells was found to be significantly inhibited by the forced expression of miR-184. *In vivo*, the growth curve of tumor xenografts showed that high expression level of miR-184 obviously slowed tumor growth. Foley *et al*. found that miR-184 ectopic overexpression in neuroblastoma cell-lines had pro-apoptotic and anti-proliferation functions through inhibiting AKT2, which was one downstream gene of the PI3K/AKT pathway [28]. Whether miR-184 inhibits glioma survival by blocking the PI3K/AKT2 pathway needs further investigation. The present study identified that miR-184 induced the apoptosis of U87 and U251 cells, increased the cell population in the G0/G1 phase, and reduced the percentage of cells in the S phase. It is reasonable to hypothesize that miR-184 inhibited the proliferation of glioma cells by increasing the percentage of early apoptotic cells, and it was suggested that miR-184 might be a novel specific biomarker for gliomas. In additional, Yuan et al. reported that miR-184 was significantly upregulated in human glioma cells. Our results differ from those by presented in the study by Yuan et al. [29]. It is reasonable to hypothesize that these differences are due to gliomas cell lines may have undergone mutations during culture.

Biological information software also predicted that miR-184 could directly target TNFAIP2 mRNA sequences at the region from 1870 to 1876. In gastric cancer and SCCHN, miR-184 bonded to the 3′-UTR of TNFAIP2, and the miR-184 binding site single nucleotide polymorphisms in TNFAIP2 contributed to tumor susceptibility [22,30]. TNFAIP2, which can be induced by treatment of TNF-α that with miR-184 can directly target TNFAIP2 in carcinoma (NPC) tissues, and is closely related to invasion and metastasis and poor survival in NPC patients [21]. A previous study demonstrated that latent membrane protein 1 (LMP1), an Epstein-Barr virus oncoprotein, induced TNFAIP2 expression via NF-κB pathway and TNFAIP2 also contribute to LMP1-induced cell motility [31]. Researchers have suggested that TNFAIP2 may play multiple roles in the development of cancer and may be particularly closely related to tumor metastasis. However, the biological function and molecular mechanism of TNFAIP2 in gliomas remain unclear. The present study is first to report that both mRNA and protein expression levels of TNFAIP2 are up-regulated in glioma cell-lines and tissues compared to non-neoplastic brain tissues. As in other reports, the results of the present study indicated that miR-184 could regulate TNFAIP2 in glioma cells. The up-regulated expression of miR-184 in U87 and U251 cells led to the obvious down-regulation of TNFAIP2 mRNA and protein expressions. Reducing the expression of miR-184 resulted in the significant up-regulation of TNFAIP2 mRNA and protein. The IHC results of the nude mice intracerebral transplantation tumors further demonstrated that U87 cells transfected with miR-184 had reduced TNFAIP2 compared to the miR-NC groups. The results confirmed that both TNFAIP2 mRNA and protein expressions were substantially down-regulated by the high expression of miR-184 in gliomas. In human gliomas, TNFAIP2 was one of the specific targets of miR-184. The study’s results showed that the expression level of TNFAIP2 was higher in human gliomas than in noncancerous brain tissues. Also, miR-184 targeted TNFAIP2 *in vitro* and *in vivo*, but it was neither elaborated upon nor proven whether the high expression of TNFAIP2 led to the process of invasion and proliferation in gliomas or whether miR-184 suppressed the survival and invasion of gliomas by down-regulating the expression of TNFAIP2. The function and mechanism of TNFAIP2 in gliomas need further investigation.

The present study demonstrated that miR-184 was markedly down-regulated in human glioma cells and tissues, TNFAIP2 was up-regulated in human glioma cells and tissues, and TNFAIP2 expression was inversely correlated with miR-184 expression. Also, the overexpression of miR-184 led to the down-regulation of TNFAIP2, and miR-184 regulated the expression of TNFAIP2 by binding to the 3′-UTR of TNFAIP2 mRNA. miR-184 had a significant suppressive effect on glioma proliferation, migration, and invasion. All the experiments showed that miR-184 was a suppressor gene in the malignant procession and carcinogenesis of gliomas and may be used to develop a miRNA-based therapeutic strategy against glioma.

## Conclusion

The results showed that miR-184 may regulate the expression of TNFAIP2 by binding to the 3′-UTR of TNFAIP2 mRNA and affecting its translation in gliomas. Thus, miR-184 inhibited the progression of gliomas and may serve as a novel therapeutic target for the treatment of gliomas.

## Additional file

Additional file 1:
**The expression of SOX2 in intracerebral transplantation tumors.**

## Abbreviations

miRNA: MicroRNAs
TNFAIP2: TNFα-induced protein 2
PML-RARs: Promyelocytic leukemia-retinoic acid receptor α
APL: Acute promyelocytic leukemia
NPC: Nasopharyngeal carcinoma
SCCHN: Squamous cell carcinoma of the head and neck
qRT-PCR: Quantitative reverse transcriptase PCR
IHC: Immunohistochemical
