# Supplementary figures and images for: MicroRNA-184 inhibits cell proliferation and invasion, and specifically targets TNFAIP2 in Glioma
Source: J Exp Clin Cancer Res. 2015 Mar 26;34(1):27. doi: 10.1186/s13046-015-0142-9 (PMC4387599; doi:10.1186/s13046-015-0142-9)

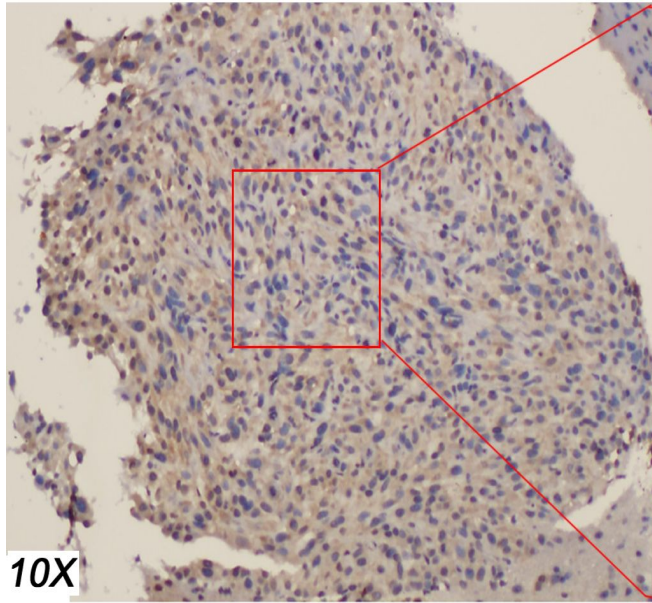

10X

SOX2

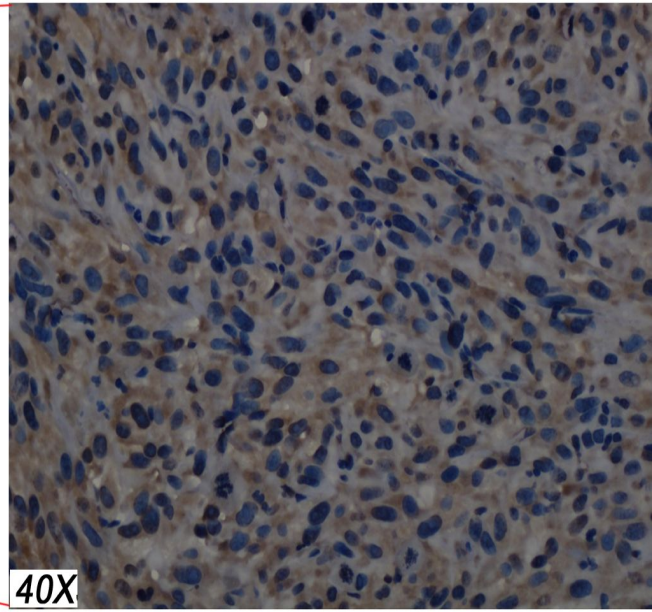

40X

SOX2

Supplement: Additional file 1: — The expression of SOX2 in intracerebral transplantation tumors. [file 13046_2015_142_MOESM1_ESM.pdf]
